# Individual‐based landscape genomics for conservation: An analysis pipeline

**DOI:** 10.1111/1755-0998.13884

**Published:** 2023-10-26

**Authors:** E. Anne Chambers, Anusha P. Bishop, Ian J. Wang

**Affiliations:** ^1^ Department of Environmental Science, Policy, and Management University of California Berkeley Berkeley California USA; ^2^ Museum of Vertebrate Zoology University of California Berkeley Berkeley California USA

**Keywords:** conservation biology, conservation genetics, genetic diversity, landscape genomics, population structure, spatial analysis

## Abstract

Landscape genomics can harness environmental and genetic data to inform conservation decisions by providing essential insights into how landscapes shape biodiversity. The massive increase in genetic data afforded by the genomic era provides exceptional resolution for answering critical conservation genetics questions. The accessibility of genomic data for non‐model systems has also enabled a shift away from population‐based sampling to individual‐based sampling, which now provides accurate and robust estimates of genetic variation that can be used to examine the spatial structure of genomic diversity, population connectivity and the nature of environmental adaptation. Nevertheless, the adoption of individual‐based sampling in conservation genetics has been slowed due, in large part, to concerns over how to apply methods developed for population‐based sampling to individual‐based sampling schemes. Here, we discuss the benefits of individual‐based sampling for conservation and describe how landscape genomic methods, paired with individual‐based sampling, can answer fundamental conservation questions. We have curated key landscape genomic methods into a user‐friendly, open‐source workflow, which we provide as a new R package, A Landscape Genomics Analysis Toolkit in R (algatr). The algatr package includes novel added functionality for all of the included methods and extensive vignettes designed with the primary goal of making landscape genomic approaches more accessible and explicitly applicable to conservation biology.

## 1 INTRODUCTION

The Anthropocene is an era of unprecedented global change, making the question of how best to conserve biodiversity more important than ever. Although broad conservation policies are typically enacted at the species level, conservation actions and management decisions regularly take place at the landscape level (Fiedler et al., 2022). Protecting species diversity and the processes that maintain it ultimately requires conserving the geographic regions that support the ecological and evolutionary processes intrinsic to population viability (Malcom & Carter, 2021). Particularly with accelerating environmental change, land‐use conversion, and habitat loss and degradation, biological conservation has increasingly become an inherently spatial problem (Shaffer et al., 2022). Uniting landscape ecology and population genetics in a single framework, landscape genetics provides a suite of spatially explicit approaches for addressing this challenge (Balkenhol et al., 2015; Keller et al., 2015; Manel et al., 2003, 2010; Segelbacher et al., 2010; Shaffer et al., 2022; Storfer et al., 2007; van Strien et al., 2014). Spatial approaches in landscape genetics involve the integration of geographic, environmental and genetic data to understand how spatial patterns of genetic variation are influenced by landscape features, environmental factors and dispersal barriers. Broadly, these include methods to delimit population boundaries, quantify the drivers of genetic differentiation, estimate landscape resistance to gene flow, characterize patterns of genetic diversity and identify genes involved in adaptation to local environmental variation (Balkenhol et al., 2015; Storfer et al., 2018; Wagner & Fortin, 2013).

Genomic data provide valuable information for identifying population boundaries, demographics and connectivity, all of which are important for conservation (Funk et al., 2012; Hohenlohe et al., 2021; Keller et al., 2015; Manel & Holderegger, 2013; Segelbacher et al., 2010). Genetic diversity itself is increasingly a target of conservation action, especially because of the role it can play in mitigating the impacts of ongoing climate change (Hoban et al., 2020; Schmidt et al., 2023). Prior to the availability and accessibility of genomic data for non‐model systems, landscape genetic studies relied on population‐based sampling, in which many individuals from each location were genotyped for a handful of genetic markers. Now that we can sequence many thousands of loci from across the genome—allowing us to capture more of the complexity that exists in natural systems (Forester & Lama, 2022; Holliday et al., 2017; Shafer et al., 2015; Supple & Shapiro, 2018)—landscape genetics has increasingly shifted towards individual‐based sampling. With genomic data, just a single individual per location can provide robust inferences about intraspecific genetic variation (Hohenlohe et al., 2021; Selmoni et al., 2020). This is advantageous for conservation efforts as well, as individual‐based sampling allows for broader geographic and environmental coverage, provides greater spatial resolution, and minimizes the overall impact of sampling on each population (Shaffer et al., 2022; Wang & Bradburd, 2014).

Despite the benefits of individual‐based sampling, a broader shift to these sampling schemes has been slowed, in large part, by methodological concerns. Landscape genetics is replete with analytical approaches, but the very breadth of those choices and the many decisions to be made in implementing each method present a certain challenge, particularly for those seeking entry into landscape genetics but even for experienced practitioners as well. Moreover, because many landscape and population genetic methods were originally designed for population‐based sampling, their validity for individual‐based sampling has sometimes come into question, further complicating the question of how best to implement a landscape genomic framework for conservation. Here, we present a pipeline for performing cutting‐edge landscape genomic analyses with individual‐based sampling, discuss the key conservation‐oriented questions it can answer and detail the considerations for each method it includes (Table 1). Our pipeline is implemented as an R package, A Landscape Genomics Analysis Toolkit in R (algatr), that is publicly available on GitHub (https://github.com/TheWangLab/algatr). algatr includes detailed walkthroughs and documentation to make it easily accessible to anyone eager to use landscape genomics to achieve actionable conservation impacts.

**TABLE 1:** Summary of key conservation‐oriented questions that our landscape genomic analysis pipeline (algatr) can answer, including input requirements for the algatr package and recent empirical examples of each method.

Question	Analysis category	Method	Input requirements	Empirical examples
How do we delineate population units for management?	Population structure	TESS (Caye et al., 2016)	Genotype dosage matrix (preferably LD‐pruned), sampling coordinates, *raster layer for mapping (optional)*	Ogbonna et al. (2021)
How is genetic variation distributed?	Genetic diversity	wingen (Bishop et al., 2023)	VCF, sampling coordinates, *raster layer for mapping (optional)*	
What are the drivers of population connectivity?	Isolation by distance and isolation by environment	MMRR (Wang, 2013)	Genetic distance matrix, environmental distance matrices or environmental layers plus sampling coordinates	Ortego et al. (2015); Sexton et al. (2016); Zhang et al. (2016)
		GDM (Ferrier et al., 2007; Fitzpatrick & Keller, 2015; Freedman et al., 2010)		Bay et al. (2018); Medina et al. (2021); Shryock et al. (2015); Wogan et al. (2020)
How can we identify and protect adaptive genetic variation?	Genotype–environment association	RDA (Capblancq & Forester, 2021)	Genotype dosage matrix, environmental layers, sampling coordinates	Forester et al. (2022)
		LFMM (Caye et al., 2019)		Carvalho et al. (2021); Cortellari et al. (2021)

## 2 INDIVIDUAL‐BASED LANDSCAPE GENOMICS FOR CONSERVATION

Having many independent loci is advantageous because each serves as an independent instantiation of the coalescent process (Leitwein et al., 2020), so sequencing large numbers of loci can provide robust estimates of genetic differentiation between localities, even when the sample size at each locus is small. For this reason, the number of loci that can be captured by next‐generation sequencing makes individual‐based sampling tenable for landscape genetics. Large genomic datasets provide strong statistical power for inferring spatial patterns of genetic variation, including the detection of genotype–environment associations (GEA), geographic barriers and corridors, and populations or genetic clusters without a priori assignments (Manel et al., 2003; Patterson et al., 2006). Individual‐based, genomic‐scale datasets also contribute distinct advantages for practical conservation biology—in particular, distributing sampling across more sites provides greater geographic and environmental coverage, better spatial resolution and lower impacts on natural populations.

### 2.1 Greater geographic and environmental coverage

For the same total sample size, individual‐based sampling schemes are able to include more localities than population‐based schemes, providing greater geographic coverage and environmental breadth. Broadening the spatial extent of a project captures greater landscape heterogeneity and more of the genetic variation across a species' range. It also allows projects to cover more areas that may be of interest for conservation and land management efforts, which is valuable for evaluating currently designated protected areas, for preserving genetic diversity and population connectivity and for assessing the potential contributions of new management areas.

Expanding coverage over environmental space provides increased power to estimate response curves that capture the relationships between a species and its environment (Shaffer et al., 2022; Wang & Bradburd, 2014), including the detection of ecologically important patterns of genetic variation and the environmental drivers of that variation (Manel et al., 2012; Selmoni et al., 2020). This increases the likelihood of capturing alleles involved in local adaptation, particularly if sampling includes environments near the edge of species' tolerance limits (Lotterhos & Whitlock, 2014; Rellstab et al., 2015; Storfer et al., 2018; Stucki et al., 2017). These alleles may play important roles in resilience to future climate change, making them important targets for conservation action. Even analyses that identify environmental drivers of neutral genetic variation can contribute important information on which environmental axes are important for maintaining adaptive potential and how environmental variation influences genetic structure and population connectivity.

### 2.2 Greater spatial resolution

Individual‐based sampling schemes also allow for greater sampling density, relative to population‐based schemes, providing finer spatial resolution for inferences of population structure, gene flow and the distribution of genetic diversity (Balkenhol & Fortin, 2015; Manel et al., 2003; Shaffer et al., 2022). By reducing the gaps between sampling localities, individual‐based sampling designs allow for more accurate identification of where population genetic elements, like genetic breaks or corridors of connectivity, occur on the landscape. By doing so, we can also understand how population genetic elements intersect with landscape features, like habitat transitions and potential physical barriers to gene flow. Homing in on where population boundaries, corridors of connectivity and regions supporting greater genetic diversity are found can assist conservation practitioners and land managers in determining which areas are most valuable for designating critical habitat and which contribute most to enabling population connectivity and persistence. This is especially important because many land management decisions take place over relatively small areas and prioritizing conservation efforts requires understanding the precise relationships between landscape elements and biodiversity.

### 2.3 Lower impact on populations

Many species are of conservation concern because they have small or heavily fragmented populations, which puts them at greater risk from population fluctuations or disturbances. In trying to understand which species or populations to prioritize for conservation efforts, researchers must strike a balance between collecting adequate sample sizes to obtain crucial information while limiting the overall impacts of sampling on population health. In the most extreme cases, population‐based sampling may not even be feasible for rare or highly imperilled species because finding sufficient numbers of individuals may prove prohibitively difficult (Supple & Shapiro, 2018). By sampling only one, or a few, individuals from each locality, individual‐based sampling minimizes the impact of sampling efforts on natural populations, conveying clear benefits for population health and sustainability and even enabling studies of some highly imperilled species that would otherwise be impossible. Sampling single individuals can also reduce the effect of repeated sampling when species management plans call for repeated genetic monitoring over time.

### 2.4 Potential downsides of individual‐based sampling

Because many population genetic analysis methods were developed for a traditional population‐based framework, their application to individual‐based sampling can present certain challenges. At a minimum, knowing which population genetic methods can be applied to individual‐based sampling requires understanding the assumptions inherent to each method, such as whether they assume normal distributions of allele frequencies per locality or whether they use population designation as an informative prior. Some methods can also be sensitive to the metrics used as inputs, and several common metrics of genetic distance and diversity cannot be calculated on an individual basis (e.g. *F*‐statistics). However, there are reliable metrics that can be calculated from individual‐based sampling that should not bias results (e.g. Shirk et al., 2017), several of which can be calculated in our pipeline (algatr).

Sampling fewer individuals per location also increases the binomial sampling noise around estimates of local allele frequencies (Wang & Bradburd, 2014). This is consequential for some methods that require precise allele frequency estimates. For example, genotype–environment association (GEA) methods were developed for population‐based sampling and may not perform as well with allele frequency estimates based on only a single individual per locality (Rellstab et al., 2015). Although not exclusive to individual‐based sampling, researchers should also be cognisant of how the spatial distribution of their sampling may affect spatial autocorrelation in their sampling design. Reflecting a common concern in spatial ecological studies, when data points have a high degree of non‐independence, analyses that do not include an autoregressive component may produce inaccurate results (Dale & Fortin, 2014; Hurlbert, 1984).

Finally, individual‐based sampling inherently limits the information acquired about the variation present within a population. This represents a reasonable trade‐off for landscape genomics, which is primarily concerned with between‐population variation, and even for conservation efforts that are concerned with landscape‐level processes, but it does present a limitation for some objectives, such as those concerned with demographic processes. For example, individual‐based sampling can only provide coarse data on population sizes and would likely not be an optimal choice for genetic monitoring of within‐population changes through time. Despite these limitations, the set of methods that have assumptions compatible with individual‐based sampling leaves researchers with many strong analytical options.

## 3 A LANDSCAPE GENOMICS PIPELINE FOR CONSERVATION

Researchers must choose from a wealth of methodological options when designing a landscape genomic study. The best path will depend on a complex set of factors, ranging from the goals and questions of the study to the assumptions and requirements for each potential method to be employed to computational costs and limitations that balance available resources and methods with dataset size. Important methodological considerations in landscape genomics begin with the broad consideration of which type of analysis to include, followed by deciding which method to perform, and then making decisions on parameter settings and options for that specific method. In addition to methodological considerations, input data must often be processed (e.g. calculating genetic or environmental distances). Understanding and prioritizing trade‐offs can be challenging even for experienced landscape geneticists and especially for conservation practitioners or other biologists who may be approaching landscape genetics for the first time. We aim to address these issues by providing a user‐friendly, accessible workflow, which is implemented in the algatr pipeline.

The algatr pipeline includes a curated set of methods that can be applied to individual‐based sampling, providing robust results under most realistic scenarios and covering the core areas of landscape genomics that are of particular interest for conservation. To make landscape genomic methodology more approachable, we have included transparent documentation in algatr for each method, including vignettes that provide step‐by‐step guidance for processing input data, running analyses, producing relevant summary statistics, interpreting results and generating figures. Although algatr makes use of (for the most part) existing packages, we have added new functionality to each of these methods and adapted, when necessary, methods to accommodate individual‐based sampling (Table S1).

Two of the main barriers to performing any analysis are ensuring input files are in the correct format and determining the proper parameter settings for respective landscape genomic methods; algatr provides functions for formatting input files and testing parameters for each method included in the pipeline. Performing any of the methods in algatr requires only a file with variant calls (i.e. a VCF file) and sampling coordinates. algatr also includes a set of utility functions that provide users with options for customization if they so desire, including functions for downloading bioclimatic data, pruning SNPs based on linkage disequilibrium, imputing missing genotypes and calculating a variety of genetic and geographic distance metrics (Table S1). We provide guidance on the application of these utility functions so that users can visualize and evaluate the effects of different ways of processing their data.

Below, we outline four key questions for conservation that landscape genomic approaches can answer and describe methods for doing so that are included in the algatr pipeline (Table 1). For each method, we describe its key components, practical considerations and relevance to conservation questions.

### 3.1 How do we delineate population units for management?

Species management plans frequently call for the designation of evolutionarily significant units, populations or genetic groups that contain some particular value to the species as a whole (Allendorf et al., 2022; Turbek et al., 2023). For example, some conservation plans target the protection of populations that harbour unique genetic variants, subspecies that exhibit geographic variation (e.g. Teixeira & Huber, 2021), or groups that maximize adaptive potential (Funk et al., 2019). A critical first step in delimiting conservation units is inferring genetic clusters based on population structure or genetic similarity.

**FIGURE 1:**
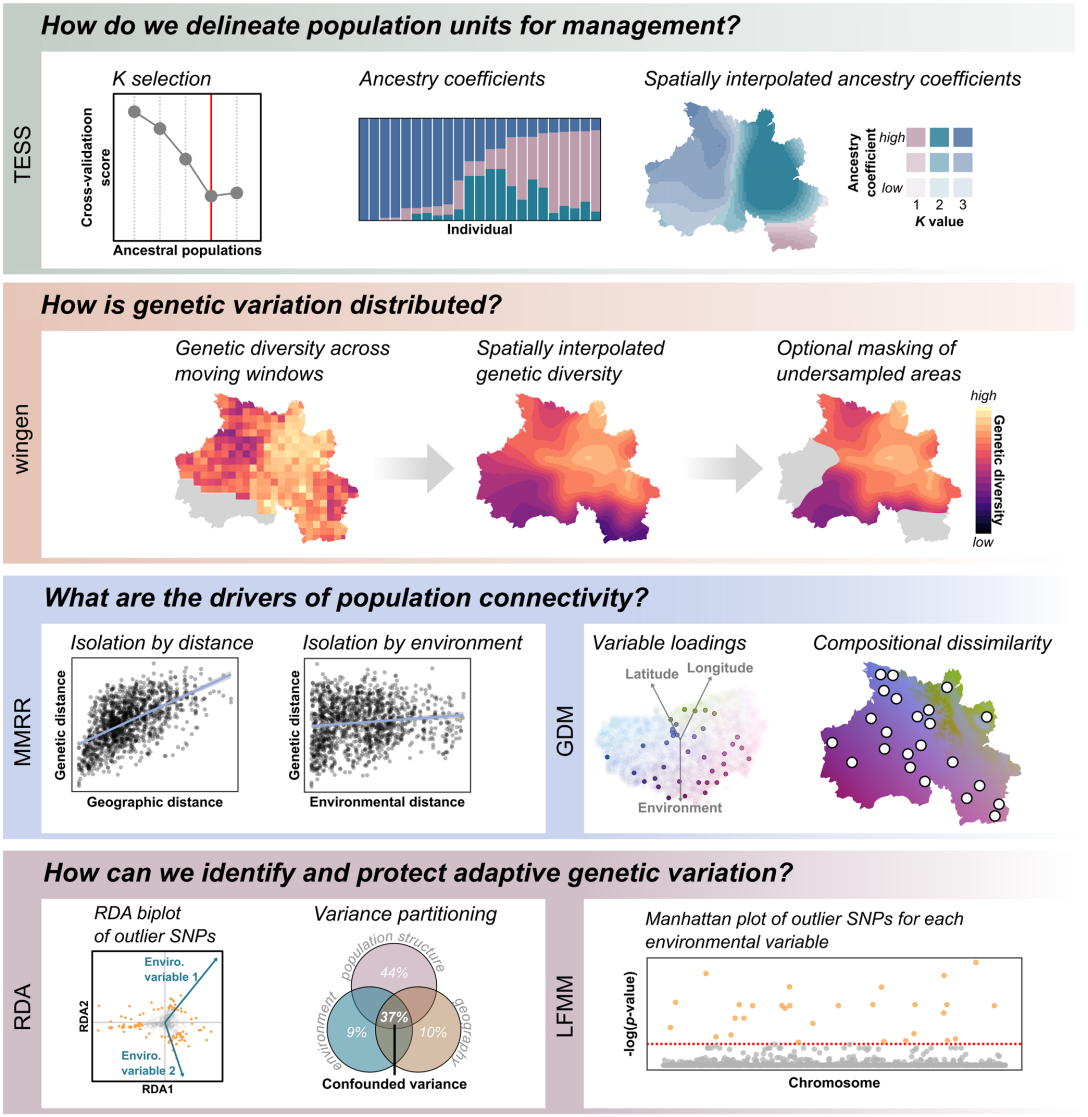
Summary of outputs produced by the algatr pipeline with suggested methods to answer conservation questions. Methods included in algatr's workflow are TESS (Caye et al., 2016), wingen (Bishop et al., 2023), multiple matrix regression with randomization (MMRR; Wang, 2013), generalized dissimilarity modelling (GDM; Ferrier et al., 2007; Fitzpatrick & Keller, 2015; Freedman et al., 2010), redundancy analysis (RDA; Capblancq & Forester, 2021) and latent factor mixed modelling (LFMM; Caye et al., 2019).

Identifying genetic clusters is straightforward when individuals are structured into discrete, isolated populations, but many species instead exhibit continuous distributions of individuals (Bradburd & Ralph, 2019; Hohenlohe et al., 2021; Manel et al., 2003). Methods to infer genetic clusters, including approaches like ADMIXTURE (Alexander et al., 2009), STRUCTURE (Pritchard et al., 2000), sNMF (Frichot et al., 2014), TESS (Caye et al., 2016) and conStruct (Bradburd et al., 2018), use clustering techniques (e.g. *K*‐means clustering) to assign individuals to genetic clusters based on how neutral genetic variation is distributed. The approaches that are most apt for landscape genomics are those that are spatially explicit, often using sample location as prior information. The algatr pipeline implements one such method, TESS (Caye et al., 2016, 2018), which infers the optimal numbers of clusters and then calculates an assignment probability or ancestry coefficient of each individual to each cluster. We have added functions to algatr to generate interpolated maps of ancestry coefficients using the *autoKrige* function from the automap package (Hiemstra et al., 2009), which differs from the interpolation done by default in TESS by producing raster maps that can then be used in downstream analyses (Figure 1).

### 3.2 How is genetic variation distributed?

Genetic diversity provides the raw material on which selection can act, allowing populations or species to respond to new threats or perturbations, ranging from novel pests and pathogens to ongoing climate change (Hoffmann et al., 2017). Loss of genetic diversity can lead to inbreeding depression, diminished adaptive potential and increased extinction risk, making it a key target for conservation action (Kardos et al., 2021; O'Grady et al., 2004). Understanding how genetic diversity is distributed across the landscape is, therefore, critical for examining the drivers of genetic variation, identifying areas that harbour greater genetic diversity and quantifying regional vulnerability to environmental change (Segelbacher et al., 2010; Sommer et al., 2013).

One challenge for individual‐based landscape genomics has been how to calculate metrics of genetic diversity, which are traditionally calculated at the population level, when samples are distributed across the landscape without discretely bounded clusters. algatr uses a recently developed method for mapping local estimates of genetic diversity based on continuous individual sampling implemented in the R package wingen (Bishop et al., 2023). wingen generates continuous maps of heterozygosity, allelic richness and nucleotide diversity using moving windows and spatial interpolation (Figure 1). wingen also includes options to account for any unevenness in sampling design using rarefaction and allows users to customize the size of the moving window and the resolution of the output maps, which are provided as rasters that can be imported for further analysis in any commonly used GIS software. These rasters thus provide detailed views of genomic diversity across the landscape that can inform land management strategies aimed at protecting genetic diversity.

### 3.3 What are the drivers of population connectivity?

One of the primary goals of systematic conservation planning is to ensure that protected areas promote population viability and persistence, and key to this is identifying how and where critical population processes occur on the landscape (Nielsen et al., 2022; Segelbacher et al., 2010). Understanding the drivers of gene flow and genetic differentiation can help conservation practitioners understand the impact of habitat fragmentation, land‐use conversion and environmental change on population connectivity (Hall & Beissinger, 2014; van Strien et al., 2014). The population connectivity between points on a landscape can diminish when gene flow is reduced over greater geographic distances, resulting in a pattern of isolation by distance (IBD; Wright, 1943), or when gene flow is restricted between regions with increasingly different environmental conditions (due to selective or non‐selective mechanisms), leading to a pattern of isolation by environment (IBE; Wang & Bradburd, 2014). The extent to which geographic and environmental isolation shape genetic divergence provides important information for maintaining functional connectivity, which is a key component of population viability, across the landscape (Segelbacher et al., 2010).

Generally, landscape genomics methods that investigate IBD and IBE rely on matrix regression, in which matrices of geographic and environmental distances serve as explanatory variables, and the response variable is a genetic distance matrix. For fitted models, the ratios between standardized regression coefficients (beta coefficients) for each explanatory variable can approximate the relative contributions of each environmental variable (or aggregated environmental distance) and geographic distance to explaining variation in genetic distances. Variable selection can be performed using backwards elimination, in which explanatory variables are incrementally removed and the model refitted until only variables with statistically significant effects remain (Ferrier et al., 2002; Wang, 2013).

algatr implements two methods for estimating IBD and IBE: generalized dissimilarity modelling (GDM; Ferrier, 2002; Ferrier et al., 2002, 2007) and multiple matrix regression with randomization (MMRR; Wang, 2013; Figure 1). GDM models turnover in the compositional dissimilarity between pairs of sites by transforming explanatory variables using a set of I‐spline basis functions (Ferrier, 2002; Ferrier et al., 2002, 2007; Fitzpatrick & Keller, 2015). GDM's main advantage is that it can account for nonlinear relationships between variables (Mokany et al., 2022). The shape of the resulting relationships can then be used to identify threshold values that may represent important areas of genetic turnover because the shape of the I‐spline functions indicates how genetic dissimilarity changes across an environmental gradient for each explanatory variable (Storfer et al., 2018). The environmental data layers used in the analysis can also be transformed based on these fitted relationships and combined into a map of genetic compositional dissimilarity across the study region (see Figure 1).

Similarly, MMRR performs linear matrix regression on genetic and environmental distances and allows for multiple independent variables (environmental and geographic distances) to be examined simultaneously (Wang, 2013). This approach provides less flexibility than GDM (Table S2) but may be less prone to overfitting, a trade‐off some researchers may prefer. MMRR performs significance testing through random permutations of the rows and columns of the dependent matrix, which is necessary because of the non‐independence of values in pairwise distance matrices. MMRR provides individual regression coefficients and *p*‐values for each explanatory variable and for the fitted model.

One remaining class of analysis in this category seeks to understand the extent to which landscape features act to restrict gene flow, a phenomenon that results in a pattern known as isolation by resistance (IBR; McRae, 2006). The workflow for investigating IBR includes first generating a resistance surface based on prior information or a hypothesis about how landscape elements contribute to differential resistance to movement for the study organism. Resistance distances can then be calculated using least‐cost path analysis (Wang et al., 2009; Wang & Shaffer, 2017) or circuit theory analysis (Dickson et al., 2019; McRae & Beier, 2007). Because the parameterization of the resistance surface is highly complex, requiring decisions that often have a significant impact on the estimation of resistance distances (Koen et al., 2012; Spear et al., 2010, 2015), and because the optimal approach to resistance surface parameterization depends strongly on the study design and objectives (Peterman et al., 2019; Spear et al., 2010; Zeller et al., 2012), algatr does not include an automated approach for generating resistance surfaces. However, if a user has generated a resistance surface with their preferred method, which could include parameterization based on habitat suitability models (Wang et al., 2008), genetic algorithms (Peterman, 2018), or gradient forest analysis of allele frequencies (Vanhove & Launey, 2023), then resistance distances can be calculated using algatr's *geo_dist* function, specifying ‘resistance’ with the type argument (type = ‘resistance’). This function uses the *commuteDistance* function in the gdistance package (van Etten & Hijmans, 2010). The resulting resistance distances can then be used in downstream analyses, including using them in MMRR or GDM to quantify IBR.

### 3.4 How can we identify and protect adaptive genetic variation?

The accessibility of genomic data for natural systems brings not only greater resolution but also opportunities to examine spatial patterns of adaptive genetic variation (Manel et al., 2010; Parisod & Holderegger, 2012; Schoville et al., 2012). Identifying ecologically important, functional genetic variation—genes that convey fitness benefits under different conditions—and the environmental forces underlying that variation provides a new dimension of valuable information for conservation efforts. Characterizing genes involved in adaptation to local environmental conditions can help guide local reintroduction efforts, assess the feasibility of genomic rescue for genetically depauperate populations, evaluate strategies for assisted gene flow, and quantify climate resilience and vulnerability (Browne et al., 2019; Frankham et al., 2014; Seaborn et al., 2021; Thurman et al., 2020).

A critical step for these objectives is to identify genes associated with specific environmental variables. Genotype–environment association (GEA) analyses quantify statistical associations between allele frequencies and environmental variables to test the hypothesis that allelic variation at a locus reflects adaptation to the local environment (Capblancq et al., 2018; Lotterhos, 2023). Because spurious genotype–environment associations could result from a variety of factors other than local adaptation, including population structure and demography, the signal of environmental selection must be parsed from the background level of neutral divergence resulting from population structure (Ahrens et al., 2018; De Mita et al., 2013; Lotterhos & Whitlock, 2014, 2015; Rellstab et al., 2015; Storfer et al., 2018). Approaches for doing so include using latent factors to represent population structure (Caye et al., 2019; Rellstab et al., 2015), conditioning on neutral variation using principal components (Duforet‐Frebourg et al., 2016), using Moran eigenvector maps to decompose spatial relationships (Forester et al., 2018), and identifying sets of loci that are putatively neutral and incorporating them as covariates in the model (Dauphin et al., 2022; Meirmans, 2015; Storfer et al., 2018).

Two GEA methods are included in algatr, latent factor mixed modelling (LFMM; Caye et al., 2019) and redundancy analysis (RDA; Figure 1). LFMM uses latent factors to account for unobserved variables, including population structure (Caye et al., 2019). This approach is advantageous because it provides controls on factors, other than selection, that may incidentally covary with allele frequencies. The challenge is determining the appropriate number of latent factors for any given dataset (called *K*‐values). algatr provides four options for doing so: a Tracy–Widom test (Frichot et al., 2013), a ‘quick elbow’ test (an approach similar to examining a scree plot), the TESS clustering algorithm (in which latent factors correspond to some measure of population structure), and K‐means clustering (Jombart et al., 2010).

RDA is a constrained ordination method that models linear, multivariate relationships (Rellstab et al., 2015). By simultaneously testing multiple loci against multiple environmental variables, RDA is able to detect multilocus selection (Forester et al., 2018; Rellstab et al., 2015) while also accounting for covariation in allele frequencies between loci. Optionally, RDA can perform variable selection using forward selection until a specified threshold is met (typically using a permutation test and adjusted *R* ^2^ values). The RDA method can also utilize covariates, such as population structure or geographic distance, alongside environmental predictors, an approach known as a partial RDA (pRDA). Finally, variance partitioning can be performed using RDA to quantify the independent contributions of each explanatory variable as well as variation that is explained by a combination of explanatory variables (i.e. confounded variance; Capblancq & Forester, 2021). algatr includes RDA and pRDA with and without variance partitioning, and it implements two approaches for determining which loci should be considered significant outliers, one that uses Z‐scores (Forester et al., 2018) and another that transforms RDA loadings into *p*‐values (Capblancq & Forester, 2021; Capblancq, Morin, et al., 2020).

### 3.5 Caveats

Like any analysis, landscape genomic methods have various limitations and assumptions, and even preparing data for these methods carries some potential pitfalls. Below, we briefly discuss some potentially important concerns that may arise during landscape genomic analysis and describe how solutions for addressing these issues can be implemented (see also Table S2). The algatr documentation aims to provide all of the information necessary to run each of the analyses and guidance on the decisions that must be made for the options offered by each method. It also provides references to several extensive and recent reviews that explore the methodology, assumptions, and theory underlying many of these methods (e.g. Capblancq, Fitzpatrick, et al., 2020; Capblancq & Forester, 2021; Fenderson et al., 2020; Forester et al., 2018; Lotterhos, 2023; Rellstab et al., 2015). The package itself also contains tools that help to evaluate different options for each method implemented in the pipeline.

GDM and MMRR take in genetic, environmental and geographic distance matrices as input—various distance metrics are available—and the choice of metric may influence downstream results. Different genetic distance metrics, in particular, may result in different outcomes. algatr provides options to calculate several metrics of genetic, geographic and environmental distances, and users should consider and test the potential impacts of different metrics on their results (Beninde et al., 2023; Shirk et al., 2017; Wang, 2020).

Several landscape genomic methods, including RDA, do not allow for any missing genotypes, meaning that studies must either remove loci containing missing data or perform imputation to fill in missing genotypes. Because removing sites containing missing data often results in greatly reduced dataset size, imputation is commonly performed to maximize data retention. Different types of imputation exist, including population structure‐based imputation (Caye et al., 2016), maximum likelihood‐based imputation (D'Angelo et al., 2010) and imputation based on the mean, median or most common genotype at each site (e.g. Capblancq & Forester, 2021), each of which introduces different assumptions (e.g. Money et al., 2015; Shi et al., 2018; Yi & Latch, 2022). To deal with missing values, algatr performs two types of imputation. The first is a per site median‐based imputation (similar to that of Capblancq & Forester, 2021), although we do not recommend this simplified approach for general use because artificially inflated *p*‐values can result if missing values are non‐random (i.e. if there is allelic bias in missing data). We have also implemented a population structure‐based imputation method that uses non‐negative matrix factorization (sNMF; Frichot et al., 2014) to assign missing values. This utilizes functions in the lea package (Frichot & François, 2015) and provides a more sophisticated (albeit computationally slower) imputation method compared with the median‐based approach. As with many similar population structure methods, it also requires a user‐selected value for *K* clusters.

Researchers should keep in mind that determining the *K‐*values that best describe their data should be done with care. This is relevant to selecting the number of latent factors for LFMM and *K* clusters for TESS. For TESS, algatr can perform manual and automatic *K‐*selection. Automatic *K‐*selection is provided mainly for simulation studies or meta‐analyses where *K*‐values have to be selected for a large quantity of datasets such that manual selection is not feasible. For LFMM, algatr implements four methods for automatically selecting the number of latent factors (also represented using the term *K*): by performing a Tracy–Widom test, a ‘quick elbow’ test, using cross‐validation scores from TESS, and *K*‐means clustering using adegenet's *find.clusters* function.

Linkage disequilibrium (LD) results in collinearity among SNPs, a pattern that can misinform landscape genomic analyses. For example, estimates of population structure can be overinflated as more SNPs appear to independently support the same pattern (Rellstab et al., 2015). This is, fortunately, fairly straightforward to address by pruning sites that are putatively in LD. A common approach involves calculating correlations between SNPs in sliding windows of custom sizes across the genome (Ahrens et al., 2018), which can be done before or after performing the GEA analysis (Capblancq & Forester, 2021). algatr performs LD‐pruning using the snprelate package (Zheng et al., 2012), which also avoids the need for phased input data, and provides options to specify window size, window overlap and the LD threshold.

## 4 CONCLUSIONS

The implementation of genetically informed conservation actions is critical to understand how best to conserve biodiversity. Landscape genomic approaches can provide important insight into how genetic variation is spatially distributed, allowing conservation practitioners to prioritize areas for conservation efforts. Genomic‐scale data have enabled a shift towards individual‐ rather than population‐based sampling because they provide more genetic resolution than was previously feasible. This shift towards individual‐based sampling reduces the impact on populations that may already be in rapid decline, while also allowing researchers to capture greater geographic and environmental coverage for their datasets and to achieve higher resolution results.

Individual‐based sampling provides a number of distinct benefits for biodiversity conservation, and landscape genomic approaches are poised to seize upon these advantages to provide actionable information for conservation and management efforts. Our R package, algatr, provides an easily accessible and user‐friendly pipeline that uses individual‐based genomic datasets to provide fine‐scale genomic and spatial resolution to answer fundamental conservation questions. Landscape genomics provides a view with ever‐increasing resolution into the processes that shape the genetic variation of Earth's biodiversity, thereby increasing our understanding of how best to protect it.

## AUTHOR CONTRIBUTIONS

All authors conceived of the study; APB and EAC wrote the algatr package, with contributions from IJW; EAC wrote and APB co‐wrote the manuscript; all authors reviewed and edited the manuscript.

## FUNDING INFORMATION

This work was supported by the California Conservation Genomics Project, with funding provided to the University of California by the State of California, State Budget Act of 2019 (UC Award ID RSI‐19‐690224) and by a National Science Foundation grant (DEB‐1845682) awarded to IJW.

## CONFLICT OF INTEREST STATEMENT

The authors declare that they have no competing interests.

## Supporting information


Table S1.
Table S2.

## Data Availability

The algatr package can be installed from GitHub: https://github.com/TheWangLab/algatr. A containerized version of the package can also be run using Docker; instructions for its installation can be found in the package's README on GitHub.
