# Supplementary material for: Individual‐based landscape genomics for conservation: An analysis pipeline
Source: Mol Ecol Resour. 2023 Oct 26;25(5):e13884. doi: 10.1111/1755-0998.13884 (PMC12142729; doi:10.1111/1755-0998.13884)
Supplement: Supplementary file 1 — Table S1. Table S2. [file MEN-25-e13884-s001.pdf]

MOLECULAR ECOLOGY

Supplemental Information for:

Individual-based landscape genomics for conservation: an analysis pipeline

E. Anne Chambers, Anusha P. Bishop, & Ian J. Wang

Table of Contents:

**Table S1**	Page 2
**Table S2**	Page 6

MOLECULAR ECOLOGY

TABLE S1 General functions and added value of the ALGATR package.

Analysis	Citation	Package	Function name	Added functionality
MMRR	Wang (2013)	N/A	*MMRR()*	First time MMRR is available in an R package
			mmrr_do_everything()	Convenience function for comprehensive MMRR analysis
			mmrr_run()	Implements automatic MMRR analysis with options for model selection
			mmrr_plot()	Convenience function for plotting fitted MMRR model and variable covariances
			mmrr_var_sel()	Implements backward elimination variable selection for MMRR
			mmrr_df()	Creates tidy data frame of model output
			mmrr_table()	Creates table of model output data frame produced by *mmrr_df()*
GDM	Ferrier et al. (2007)	GDM (Fitzpatrick et al., 2022)	*gdm_do_everything()*	Convenience function for comprehensive GDM analysis
			gdm_run()	Implements automatic GDM analysis with options for resistance and topographic distances (in place of Euclidean geographic distances)
			gdm_var_sel()	Implements variable selection for GDM
			gdm_df()	Creates tidy data frame of model output including calculation of coefficients from I-splines
			gdm_map()	Produces GDM map of projected dissimilarity
			gdm_plot_vars()	Produces GDM PCA plot which serves as a “legend” for the GDM map
			gdm_plot_diss()	Produces two compositional dissimilarity plots: one with the observed response data against raw ecological distance from the model, and the other with the observed response against the predicted response from the model (after link function is applied)
			gdm_plot_isplines()	Produces I-spline plots for each variable included in GDM model

			gdm_table()	Creates table of model output data frame produced by *gdm_df()*
LFMM	Caye et al. (2019)	LFMM (Jumentier, 2021)	*lfmm_do_everything()*	Convenience function for comprehensive LFMM analysis
			lfmm_run()	Implements automatic LFMM analysis with options for outlier detection and p-value correction
			select_K()	Multiple methods for K selection, both manual and automatic
			lfmm_df()	Creates tidy data frame of LFMM results
			lfmm_table()	Creates table of LFMM data frame produced by *lfmm_df()* with options for filtering and ordering data
			lfmm_qqplot()	Produces quantile-quantile (qq) plot with LFMM results (theoretical quantiles against *p*-value quantiles)
			lfmm_manhattanplot()	Convenient production of Manhattan plot from LFMM results
RDA	Lasky et al. (2012); Forester et al. (2016); Forester et al. (2018); Capblancq & Forester (2021)	Code adapted from Capblancq & Forester (2021)	*rda_do_everything()*	Convenience function for comprehensive RDA analysis
			rda_run()	Convenient implementation of both basic and partial RDA analyses with options for geographic and PC-based correction
			rda_getsnps()	Convenient implementation of both *p*-value[†] and Z-score[‡] outlier identification methods
			rda_plot()	Convenient production of Manhattan plots, RDA biplots, and RDA histograms
			rda_cor()	Creates tidy dataframe of correlations between significant SNPs and environmental variables
			rda_table()	Creates table of correlation dataframe produced by *rda_cor()* with options for filtering and ordering data
			rda_varpart()	Variance partitioning of partial RDAs
			rda_varpart_table()	Creates table of variance partitioning results

MOLECULAR ECOLOGY

TESS	Caye et al. (2016)	TESS3R (Caye et al., 2016)	*tess_do_everything()*	Convenience function for comprehensive TESS analysis
			tess_ktest()	Convenience function for testing multiple *K* values with manual and automatic *K* selection
			tess_krig()	Produces kriged Q matrix rasters using automap *autoKrige()* function
			tess_barplot()	Produces stacked barplot with Q matrix values for each individual, colorized by selected *K* with options for filtering and ordering data
			tess_ggbarplot()	Produces ggplot2 bar plots of Q matrix values
			tess_ggplot()	Produces plots of kriged Q matrix values with multiple options for customizability
			tess_plot_allK()	Produces separate plots of kriged Q matrix values for each *K*
wingen	Bishop et al. (2023)	WINGEN (Bishop et al., 2023)	*wingen_do_everything()*	Convenience function for comprehensive wingen analysis
General utilities			*simple_impute()*	Convenience function for simple imputation (to the median) of missing genotype values
			str_impute()	Convenience function for imputing based on population structure
			snmf_bestK()	Convenience function to select “best” *K* based on minimizing cross-entropy criteria from sNMF results
			geno_to_dosage	Convenience function to convert matrix of type LFMM or geno to dosage matrix
			gen_to_geno()	Convenience function to convert genotype dosage matrix or VCF to geno type object
Data processing utilities			*ld_prune()*	Convenience function for pruning sites in linkage disequilibrium
Environmental and geographic data utilities			*geo_dist()*	Convenience function for calculating Euclidean, topographic, or resistance distances from coordinates
			env_dist()	Convenience function for calculating environmental distances

MOLECULAR ECOLOGY

	get_worldclim()	Convenience function to automatically download, merge, and crop WorldClim tiles based on sample coordinates
	check_env()	Convenience function to check for collinearity between environmental layers
	check_vals()	Convenience function to check for collinearity between extracted values
	check_dists()	Convenience function to check for collinearity between geographic and environmental distances
Genetic distances utilities	*gen_dist()*	Convenience function for calculating Euclidean, Bray-Curtis, proportion of shared alleles, and PC-based genetic distances from sequence data and processing Plink distances
	gen_dist_corr()	Creates figure to compare genetic distance metrics
	gen_dist_hm()	Creates heatmap of genetic distances
Masking utilities	*rm_islands()*	Removes islands from environmental layers
	extrap_mask()	Masks rasters based on (1) environmental range of coordinates, (2) areas outside the mean +/- SD*NSD of any of the values of the coordinates, (3) a circular buffer around each coordinate, or (4) a convex hull around the coordinates

[†]The *p*-value method is described in Capblancq et al. (2020) and Capblancq and Forester (2021).
[‡]The Z-score method is described in Forester et al. (2018).

MOLECULAR ECOLOGY

TABLE S2 Main considerations and caveats for each method in the ALGATR package, including a citation for further information, if applicable.

Method	Consideration/caveat	Further information
TESS	Users must determine how to prune data to account for linkage disequilibrium prior to running TESS	Caye et al., 2016
TESS	*K* selection required; no straightforward test for determining "optimal" *K*	Caye et al., 2016
TESS	Option to set the spatial regularization parameter (depending on suspected spatial autocorrelation between sampling localities) and choose between a projected least squares algorithm or an alternating quadratic programming algorithm	Caye et al., 2016, 2018
MMRR	Fits only linear relationships between genetic and environmental/geographic distances	Wang, 2013
MMRR, GDM	Correlations between predictor variables can lead to statistical suppression, resulting in unreliable coefficient estimates	
GDM	By default, GDM fits three I-splines with knots at the minimum, median, and maximum quantiles; however, the number of splines and knots can be changed to modify the shape of the fit	Ferrier et al., 2007; Fitzpatrick & Keller, 2015; Freedman et al., 2010
RDA	The selected ordination method (i.e., simple vs partial RDA) can impact which SNPs are identified as outliers; if running a partial RDA, users must determine which variables should be considered covariates in the model (e.g., population structure)	Forester et al., 2018; Capblancq et al., 2021
LFMM	The number of latent factors must be selected; no straightforward test for determining "optimal" number of latent factors	Caye et al., 2019
wingen	Window size and raster spatial scale must be set by the user	Bishop et al., 2023
